# An analysis of DPV and DIVE registry patients with chronic kidney disease according to the finerenone phase III clinical trial selection criteria

**DOI:** 10.1186/s12933-023-01840-5

**Published:** 2023-05-08

**Authors:** Peter Bramlage, Stefanie Lanzinger, Steffen Mühldorfer, Karsten Milek, Anton Gillessen, Roman Veith, Tobias Ohde, Thomas Danne, Reinhard W. Holl, Jochen Seufert

**Affiliations:** 1 Institute for Pharmacology and Preventive Medicine, Bahnhofstrasse 20, 49661 Cloppenburg, Germany; 2 Institut für Epidemiologie und medizinische Biometrie, ZIBMT; Universität Ulm, Ulm, Germany; 3 Deutsches Zentrum für Diabetesforschung e.V., Munich-Neuherberg, Germany; 4 Darm-Und Pankreaskarzinomzentrum, Klinikum Bayreuth, Bayreuth, Germany; 5 Diabetologische Schwerpunktpraxis, Hohenmölsen, Germany; 6 Innere Medizin, Herz-Jesu-Krankenhaus, Münster, Germany; 7 Nephrologie, Klinikum Bad Hersfeld, Bad Hersfeld, Germany; 8 Diabeteszentrum Essen-Nord, Essen, Germany; 9 Kinderkrankenhaus auf der Bult, Diabeteszentrum für Kinder und Jugendliche, Hannover, Germany; 10 Abteilung Endokrinologie und Diabetologie, Klinik für Innere Medizin II, Universitätsklinikum Freiburg, Medizinische Fakultät, Freiburg, Germany

**Keywords:** Diabetes, Diabetic kidney disease, Hypertension, Chronic kidney disease, Glomerular filtration rate, Albuminuria, Diagnostics, Pharmacotherapy

## Abstract

**Background:**

The FIDELIO-DKD and FIGARO-DKD randomized clinical trials (RCTs) showed finerenone, a novel non-steroidal mineralocorticoid receptor antagonist (MRA), reduced the risk of renal and cardiovascular events in patients with type 2 diabetes mellitus (T2DM) and chronic kidney disease (CKD). Using RCT inclusion and exclusion criteria, we analyzed the RCT coverage for patients with T2DM and CKD in routine clinical practice in Germany.

**Methods:**

German patients from the DPV/DIVE registries who were ≥ 18 years, had T2DM and CKD (an estimated glomerular filtration rate [eGFR] < 60 mL/min/1.73 m^2^ *OR* eGFR ≥ 60 mL/min/1.73m^2^ and albuminuria [≥ 30 mg/g]) were included. RCT inclusion and exclusion criteria were then applied, and the characteristics of the two populations compared.

**Results:**

Overall, 65,168 patients with T2DM and CKD were identified from DPV/DIVE. Key findings were (1) Registry patients with CKD were older, less often male, and had a lower eGFR, but more were normoalbuminuric vs the RCTs. Cardiovascular disease burden was higher in the RCTs; diabetic neuropathy, lipid metabolism disorders, and peripheral arterial disease were more frequent in the registry. CKD-specific drugs (e.g., angiotensin-converting enzyme inhibitors [ACEi] and angiotensin receptor blocker [ARBs]) were used less often in clinical practice; (2) Due to the RCT’s albuminuric G1/2 to G4 CKD focus, they did not cover 28,147 (43.2%) normoalbuminuric registry patients, 4,519 (6.9%) albuminuric patients with eGFR < 25, and 6,565 (10.1%) patients with microalbuminuria but normal GFR (≥ 90 ml/min); 3) As RCTs required baseline ACEi or ARB treatment, the number of comparable registry patients was reduced to 28,359. Of these, only 12,322 (43.5%) registry patients fulfilled all trial inclusion and exclusion criteria. Registry patients that would have been eligible for the RCTs were more often male, had higher eGFR values, higher rates of albuminuria, more received metformin, and more SGLT-2 inhibitors than patients that would not be eligible.

**Conclusions:**

Certain patient subgroups, especially non-albuminuric CKD-patients, were not included in the RCTs. Although recommended by guidelines, there was an undertreatment of CKD-patients with renin-angiotensin system (RAS) blockers. Further research into patients with normoalbuminuric CKD and a wider prescription of RAS blocking agents for CKD patients in clinical practice appears warranted.

**Supplementary Information:**

The online version contains supplementary material available at 10.1186/s12933-023-01840-5.

## Background

The prevalence of chronic kidney disease (CKD) has increased in recent decades alongside an increase in diabetes and hypertension, the main drivers of CKD [1]. Kidney disease, attributable to diabetes mellitus (diabetic kidney disease; DKD), is one of the most common complications of diabetes and affects ~ 40% of patients with type 2 diabetes (T2DM) [2, 3]. The typical presentation of DKD includes a long-standing duration of diabetes, retinopathy, albuminuria without hematuria and gradually progressive kidney disease. However, reduced eGFR without albuminuria has been also frequently reported and is becoming increasingly more prevalent. DKD can ultimately lead to end-stage renal disease and is associated with an increased risk of cardiovascular (CV) disease and death [4–6]. Finally, people with diabetes can also develop CKD due to etiologies other than diabetes and some may have a combination of DKD and non-diabetic CKD [7].

Renin-angiotensin system (RAS) blockers, such as angiotensin converting enzyme inhibitors (ACEis) and angiotensin receptor blockers (ARBs), in the context of a multifactorial risk minimization strategy have been tested for their ability to revert CKD progression or at least prevent its further deterioration and were included in guidelines as a standard therapy for diabetic patients with CKD [8–12]. Furthermore, recent studies have shown that sodium-glucose co-transporter-2 (SGLT2) inhibitors improve renal outcomes [12–14]. However, despite the use of ACEIs, ARBs, and the concomitant use of SGLT2 inhibitors, the rate of renal deterioration remains high, with more than twice the normal decline in kidney function in patients with diabetes compared to patients without diabetes [13].

One of the potential reasons for the residual renal deterioration is an increase in aldosterone levels which are elevated in up to 50% of patients treated with RAS blockers within a year of initiating treatment, leading to increases in albuminuria and impairment of kidney function [15]. In line with this assumption, it has been shown that mineralocorticoid receptor antagonists (MRAs), even when added to ACEIs and ARBs, decrease proteinuria in patients with CKD [16]. As earlier MRAs, such as spironolactone and eplerenone, were effective but tended to show intolerable side effects, such as gynecomastia and hyperkalemia, a more tolerable but at least as effective and safe next-generation MRA was developed. Finerenone, a novel, non-steroidal, selective MRA was investigated in two large phase III clinical trials in patients with CKD [17, 18]. In FIDELIO-DKD, finerenone improved renal outcomes in T2DM patients with advanced CKD [19]. In FIGARO-DKD, finerenone improved CV outcomes in T2DM patients with early CKD [20]. The outcomes of the two studies were confirmed in the pooled FIDELITY analysis [21].

We were interested in the coverage of these two clinical trials for T2DM patients with CKD in Germany. As we recently published data on the prevalence of CKD [22] and the degree of guideline-conformant treatment [23], we now applied the inclusion and exclusion criteria used for the FIDELITY trial [21] to CKD patients in the registry. In our analysis, we explored:

A) Differences between the CKD population documented in DPV/DIVE and the population that was part of the combined finerenone clinical trials (FIDELITY).
B) The coverage of the FIDELITY CKD population focusing on albuminuric patients compared with CKD patients included in the registry (DPV and DIVE).
C) The impact of the baseline requirement of the prescription of ACEi or ARBs for the study population to be in line with guideline-recommendations AND the coverage of the FIDELITY population for patients with CKD within DPV/DIVE when all the CKD criteria, the requirement for ACEi/ARB treatment at baseline, and all the further inclusion and exclusion criteria were applied.

## Methods

### Study design and data sources

This analysis used combined data from the DPV and DIVE registries [24–26]. In short, the DPV initiative collects data on patients with diabetes mellitus from centers predominantly in Germany. Data are collected using DPV software and the anonymized data are sent to the University of Ulm for aggregation into the database. The DPV initiative was established in 1995 and was approved by the ethics committee of the University of Ulm. Data collection was further approved by local review boards.

The DIVE registry was established in 2011 [27]. Consecutive patients with diabetes mellitus, regardless of their disease stage, were enrolled from specialized diabetes centers across the country, and continue to be followed up. Data are entered into the DPV online database using DPV software. The protocol was approved by the ethics committee of Hannover Medical School, and all patients provided written informed consent.

### Patients and centers

A total of 216 specialized diabetes-centers from Germany were included in the present analysis. Patients were sampled in March 2022 and included in the current analysis if they had T2DM, were at least 18 years old, initially registered from 2015 to 2021, and had a clinical diagnosis of CKD [28, 29].

### Documentation

For the current analysis, data regarding age, gender, body mass index (BMI), blood pressure, renal parameters, antidiabetic and antihypertensive drug treatment, and current comorbidities were considered. Data for the most recent treatment year per patient were aggregated and analyzed. For retinopathy, the two most recent treatment years were considered. eGFR was calculated according to the Chronic Kidney Disease Epidemiology Collaboration (CKD-EPI) formula [30].

### Statistics

Data from all patients documented at German sites in DPV and DIVE were combined and analyzed as a single data set. Patients were selected based on the availability of required clinical data, while patients with missing information on other values were excluded and the respective number of patients with missing information reported in the demographics table. CKD was defined as eGFR < 60 mL/min/1.73 m^2^ *OR* eGFR ≥ 60 mL/min/1.73m^2^ and albuminuria (≥ 30 mg/g) [28, 29]. Table 1 describes the patient selection based on the trials’ CKD criteria. Table 2 summarizes the selection criteria (inclusion and exclusion) used for the FIDELIO-DKD/FIGARO-DKD randomized clinical trials (RCTs) [17, 18]. In general, similar inclusion/exclusion criteria could be applied to the DPV/DIVE population, apart from a few minor discrepancies due to the type of information recorded in the registry database. It is important to note, however, that diabetic retinopathy and heart failure had specific definitions in the clinical trials that could not completely be reflected in the registry dataset.

**Table 1:** CKD definitions used in FIDELIO-DKD vs. FIGARO-DKD clinical trials [17, 18]

FIDELIO-DKD	FIGARO-DKD	Difference	DPV/DIVE Analysis
Patients with a clinical diagnosis of CKD based on either of the following criteria at the run-in and screening visits:	Patients with a clinical diagnosis of CKD based on either of the following criteria at the run-in and screening visits:		
Patient is diagnosed with Persistent high albuminuria by following factors:- UACR ≥30 mg/g (≥3.4 mg/mmol) but <300 mg/g (<33.9 mg/mmol)- CKD-EPI showing eGFR ≥25 but <60 mL/min/1.73 m2 with the history of diabetic rectinopathy.	Patient is diagnosed with Persistent high albuminuria by following factors:- UACR ≥30 mg/g (≥3.4 mg/mmol) but <300 mg/g (<33.9 mg/mmol)- CKD-EPI showing eGFR ≥25 but ≤90 mL/min/1.73 m2 in the medical history	In FIDELIO-DKD in 10% of the patients with microalbuminuria, eGFR ≥ 60 mL/min/1.73 m^2^ (CKD-EPI) a retinopathy was required for inclusion	All patients that fulfilled either criteria were included, retinopathy was disregarded
OR	OR		
Patient is diagnosed with Persistent very high albuminuria by following factors:- UACR ≥300 mg/g (≥33.9 mg/mmol)- CKD-EPI showing eGFR ≥25 but <75 mL/min/1.73 m2	Patient is diagnosed with Persistent very high albuminuria by following factors:- UACR ≥300 mg/g (≥33.9 mg/mmol)- CKD-EPI showing eGFR ≥60 mL/min/1.73 m2	In FIDELIO-DKD no patients with macroalbuminuria, and eGFR ≥ 75 mL/min/1.73 m^2^ (CKD-EPI) were included In FIGARO-DKD no patients with macroalbuminuria but eGFR < 60 mL/min/1.73 m^2^ (CKD-EPI) were included	All patients that fulfilled either criteria were included

*CKD* chronic kidney disease; *CKD-EPI* Chronic Kidney Disease Epidemiology Collaboration; *DKD* diabetic kidney disease; *eGFR* estimated glomerular filtration rate; *UACR* urinary albumin-to creatinine ratio

**Table 2:** Selection criteria for FIDELIO-DKD/FIGARO-DKD [17, 18] and how they apply to the DPV/DIVE CKD population (n = 65,168)

	FIDELIO-DKD / FIGARO-DKD	Eligible DPV/DIVE patients	Ineligible DPV/DIVE patients
Inclusion criteria	Men or women aged 18 years and older	Identical	Identical
	Women of child-bearing potential with a negative pregnancy test and agreeing to use adequate contraception	Not available	Not available
	Patients with T2DM as defined by the American Diabetes Association	Identical	Identical
	Patients with a clinical diagnosis of CKD (see Table 1)	Identical	Identical
	Prior treatment with ACEIs or ARBs	Identical	Excluded
	Serum potassium ≤ 4.8 mmol/L at both the run-in and screening visits	Identical	Excluded
Exclusion criteria			
*Medical and surgical history*	Known significant non-diabetic renal disease, including clinically relevant renal artery stenosis	Excluded (based on Analgesic nephropathy, IgA nephropathy, Lithium, Lupus nephritis, xanthine oxidase deficiency, chemotherapy toxicity)	Included
	UACR > 5,000 mg/g (> 565 mg/mmol) at the run-in or screening visit	Excluded^a^ (based on the median of the last year)	Included
	Glycosylated hemoglobin > 12% (> 108 mmol/mol) at the run-in or screening visit	Excluded^a^ (based on the median of the last year)	Included
	Uncontrolled arterial hypertension with mean sitting SBP ≥ 170 mmHg or mean sitting DBP ≥ 110 mmHg at the run-in visit or mean sitting SBP ≥ 160 mmHg or mean sitting DBP ≥ 100 mmHg at the screening visit	Excluded^a^ (based on the median of the last year)	Included
	SBP < 90 mmHg at the run-in or screening visit	Excluded^a^ (based on the median of the last year)	Included
	Patients with a clinical diagnosis of chronic heart failure with reduced ejection fraction and persistent symptoms (New York Heart Association class II–IV) at the run–in visit	Excluded: Patients with a clinical diagnosis of chronic heart failure (no discrimination between reduced and preserved EF)	Included
	Stroke, transient ischemic cerebral attack, acute coronary syndrome, or hospitalization for worsening heart failure, in the 30 days before the screening visit	Excluded	Included
	Dialysis for acute renal failure in the 12 weeks before the run-in visit	Excluded	Included
	Renal allograft in place or a scheduled kidney transplant in the 12 months after the run-in visit	Excluded	Included
	Addison’s disease	Excluded	Included
	Hepatic insufficiency classified as Child–Pugh C	Excluded	Included
*Medication and drug use*	Concomitant therapy with eplerenone, spironolactone, any renin inhibitor, or potassium-sparing diuretic which cannot be discontinued at least 4 weeks before the screening visit	Excluded: Potassium sparing: Amiloride, Triamterene, spironolactone and eplerenone	Included
	Concomitant therapy with both ACEIs and ARBs which cannot be discontinued for the purpose of the studies	No information on the ability to discontinue ACEi/ARB	Included
	Concomitant therapy with potent cytochrome P450 isoenzyme 3A4 inhibitors or inducers (to be stopped at least 7 days before randomization)	Excluded	Included
*Other*	Any other condition or therapy which would make the patient unsuitable for the studies and would not allow participation for the full planned study period (e.g., active malignancy or other condition limiting life expectancy to < 12 months)	Excluded: Active malignancy or other condition limiting life expectancy to < 12 months	Included
	Pregnant or breast-feeding or intention to become pregnant during the studies	Not available	Included
Not applicable	Written, informed consent signed before any study-specific procedure	Not applicable	Not applicable
	Known hypersensitivity to study tx (active substance or excipients)	Not applicable	Not applicable
	Previous assignment to treatment during the studies	Not applicable	Not applicable
	Previous (within 30 days before randomization) or concomitant participation in another clinical study (i.e., Phase I–III clinical studies) with investigational medicinal product, except for participation in the run-in and screening periods of Studies 17,530 and 16,244	Not applicable	Not applicable
	Close affiliation with the investigational site: for example, a close relative of the investigator or dependent person (e.g., employee or student of the investigational site)	Not applicable	Not applicable

*ACEI* angiotensin-converting enzyme inhibitor; *ARB* angiotensin receptor blocker; *CKD-EPI* Chronic Kidney Disease Epidemiology Collaboration; *DBP* diastolic blood pressure; *DKD* diabetic kidney disease; *eGFR* estimated glomerular filtration rate; *EF* ejection fraction; *SBP* systolic blood pressure; *T2DM* type 2 diabetes mellitus; *UACR* urinary albumin-to-creatinine ratio

^a^There is no run-in or screening visit in DPV/DIVE so it was assumed to be equivalent to the last visit (cross-section)

Categorical variables are presented as percentages. Continuous variables are presented as means with standard deviations or medians with first and third quartiles (Q1, Q3). Unadjusted comparisons were conducted using a Chi-squared or Kruskal–Wallis test. The false discovery rate method was used to correct p-values for multiple testing. A two-sided p-value < 0.05 was considered statistically significant. Statistical analysis was performed using SAS version 9.4 (build TS1M7).

## Results

**Fig. 1:**
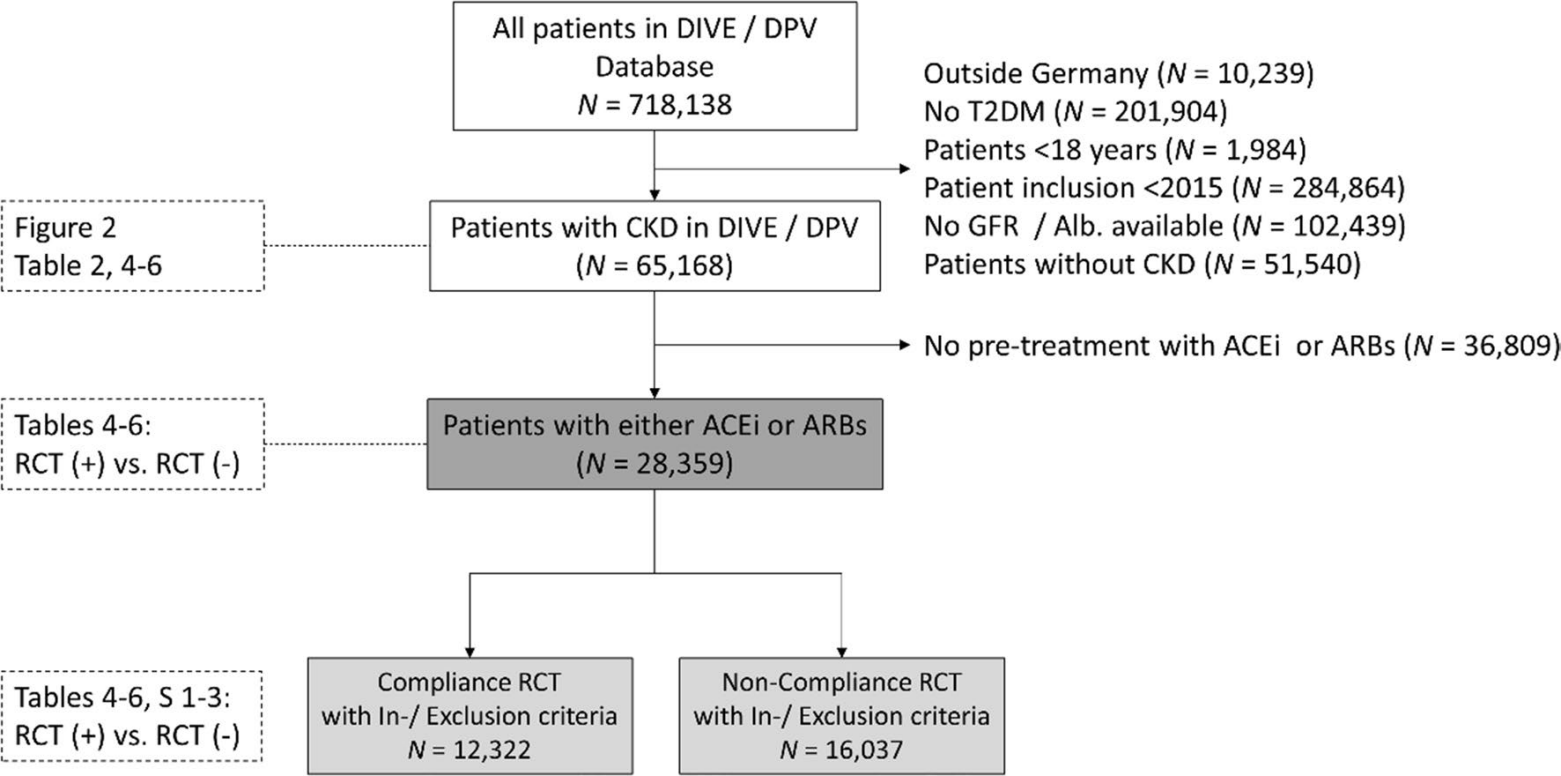
Patient population. *CKD* chronic kidney disease (defined as eGFR < 60 mL/min/1.73 m^2^ or eGFR ≥ 60 mL/min/1.73 m.^2^ and albuminuria ≥ 30 mg/g; *RCT* randomized controlled trial; T2DM, type-2 diabetes mellitus [28, 29]

A total of 65,168 adult patients with T2DM and CKD from Germany were included in the current analysis. Reasons for patient exclusion from the analysis are displayed in Fig. 1 and comprised patients residing outside of Germany, patients with forms of diabetes other than T2DM, patients aged < 18 years, patients included before 2015, missing data for GFR/albuminuria, and patients presenting without CKD.

Compliance and non-compliance with the RCT criteria were based on the inclusion and exclusion criteria as outlined in Table 2.

### Comparison of patients in FIDELITY vs. DPV/DIVE

A comparison of patient characteristics between the 65,168 registry patients and the FIDELITY population is presented in Table 3 (*columns 1 and 2*). CKD patients in the registry were older (72.6 vs. 64.8 years), less often male (52.6 vs. 69.8%), had a shorter duration of diabetes (13.7 vs. 15.4 years), had a lower eGFR (54.2 vs. 57.6 mL/min/1.73 m^2^), and were more often normoalbuminuric (43.2 vs. 1.8% with UACR < 30 mg/g) compared with patients included in the RCTs.

**Table 3:** General patient characteristics

	Meta-analysis	DPV/DIVE patients with CKD^a^				
	FIDELITY	Total	Total ACEi/ARB	RCT ( +)	RCT (-)	p-value
	n = 13,026	n = 65,168	n = 28,359	n = 12,322	n = 16,037	RCT + vs. -
Age, years, mean ± SD	64.8 ± 9.5	72.6 ± 12.1	73.4 ± 11.3	72.9 ± 10.6	73.7 ± 11.9	< 0.0001
Gender, male, %	69.8	52.6	52.6	57.2	49.1	< 0.0001
BMI, kg/m^2^, mean ± SD	31.3 ± 6.0	31.2 ± 6.9	31.5 ± 6.8	31.5 ± 6.7	31.5 ± 6.9	0.7183
Duration of diabetes, years, mean ± SD	15.4 ± 8.7	13.7 ± 10.0	13.9 ± 9.9	13.8 ± 9.9	14.0 ± 9.9	0.0423
Systolic blood pressure, mmHg, mean ± SD	136.7 ± 14.2	135.3 ± 18.7	136.6 ± 19.1	138.7 ± 19.2	135.0 ± 18.8	< 0.0001
Diastolic blood pressure, mmHg, mean ± SD	76.4 ± 9.6	75.8 ± 10.8	76.0 ± 11.0	77.0 ± 11.0	75.3 ± 10.9	< 0.0001
eGFR (CKD-EPI), mL/min/1.73 m^2^, mean ± SD	57.6 ± 21.7	54.2 ± 25.1	53.7 ± 24.0	60.1 ± 19.5	48.6 ± 26.0	< 0.0001
≥ 60, %	39.9	31.3	30.8	49.8	15.8	< 0.0001
45 to < 60, %	26.4	29.1	29.6	23.4	34.5	< 0.0001
25 to < 45, %	32.5	29.1	29.9	26.9	32.3	< 0.0001
< 25, %	1.2	12.7	11.3	0.0	20.0	< 0.0001
UACR, mg/g						
< 30, %	1.8	43.2	41.5	0.0	73.4	< 0.0001
30 to < 300, %	31.5	43.4	43.9	75.1	19.9	< 0.0001
≥ 300, %	66.7	13.4	14.6	24.9	6.7	< 0.0001
Serum potassium, mEq/L, mean ± SD	4.35 ± 0.44	4.39 ± 0.66^b^	4.41 ± 0.66^c^	4.4 ± 0.6	4.5 ± 0.7	< 0.0001
HbA1c, %, mean (SD)	7.7 ± 1.4	7.6 ± 1.9	7.6 ± 1.9	7.7 ± 1.9	7.6 ± 1.8	< 0.0001

FIDELITY [21] was a pooled analysis of FIDELIO-DKD and FIGARO-DKD [19, 20]

*BMI* body mass index; *CKD-EPI* Chronic Kidney Disease Epidemiology Collaboration; *eGFR* estimated glomerular filtration rate; *IQR* interquartile range; *RCT* randomized controlled trial; *UACR* urinary albumin-to-creatinine ratio

^a^The difference between the DPV/DIVE total and the combined RCT ± groups is the results of 36,809 patients that did not receive either ACEi or ARB at baseline

^b^Available for 17,584 patients

^c^Available for 10,022 patients

In addition, there were several differences between the registry and the FIDELITY population with respect to their comorbid disease profile (Table 4): On the one hand, patients in the registry had lower rates of pre-existent CV disease, such as hypertension (78.0 vs. 96.5%), diabetic retinopathy (6.0 vs. 38.0%), coronary artery disease (CAD; 11.0 vs. 30.7%), myocardial infarction (10.2 vs. 15.5%), and ischemic stroke (9.0 vs. 11.9%). On the other hand, higher rates of diabetic neuropathy (45.9 vs. 26.9%), lipid metabolism disorders (87.4 vs. 45.6%), peripheral arterial disease (23.8 vs. 16.0%), and heart failure (12.3 vs. 7.5%) were observed in the registry population.

**Table 4:** Comorbidity

	Meta-analysis	DPV/DIVE patients with CKD^a^				
	FIDELITY	Total	Total ACEi/ARB	RCT ( +)	RCT (-)	p-value
	n = 13,026	n = 65,168	n = 28,359	n = 12,322	n = 16,037	RCT + vs. -
Arterial hypertension, n (%)	96.5	78.0 (140/90)	100.0	100.0	100.0	1.0
Diabetic retinopathy, n (%)	38.0	6.0	7.5	7.5	7.5	0.9981
Diabetic neuropathy, n (%)	26.9	45.9	50.5	53.7	48.0	< 0.0001
Lipid metabolism disorders, n (%)	45.6	87.4	91.6	92.0	91.2	0.0320
History of CV disease, n (%)						
Coronary artery disease w/o MI, n (%)	30.7	11.0	13.9	13.2	14.5	< 0.0001
Peripheral arterial disease, n (%)	16.0	23.8	27.2	27.5	27.0	0.3972
Myocardial infarction, n (%)	15.5	10.2	13.0	12.3	13.5	0.0048
Ischemic stroke, n (%)	11.9	9.0	11.7	11.6	11.7	0.8986
Heart failure, n (%)	7.5	12.3	16.6	15.3	17.7	< 0.0001

FIDELITY [21] was a pooled analysis of FIDELIO-DKD and FIGARO-DKD [19, 20]

*CV* cardiovascular; *RCT* randomized controlled trial

^a^The difference between the DPV/DIVE total and the combined RCT ± groups is the results of 36,809 patients that did not receive either an ACEi or ARB at baseline

The proportion of patients in the registry receiving concomitant medication was substantially lower than the corresponding proportion in the FIDELITY population (Table 5), including statins (36.9 vs. 72.2%), platelet aggregation inhibitors (9.8 vs. 56.0%), antihypertensive drugs (ACEi 26.8 vs. 39.0%; ARBs 16.7 vs. 60.9%; beta-blockers 36.3 vs 49.9%; loop diuretics 14.1 vs. 21.5; thiazide diuretics 4.6 vs. 24.2%; and calcium antagonists 20.3 vs. 56.5%), and glucose-lowering drugs (metformin 38.0 vs 58.0% and sulfonylurea 5.2 vs. 26.0%).

**Table 5:** Concomitant drug treatment

	Meta-analysis	DPV/DIVE patients with CKD^a^				
	FIDELITY	Total	Total ACEi/ARB	RCT ( +)	RCT (-)	p-value
	n = 13,026	n = 65,168	n = 28,359	n = 12,322	n = 16,037	RCT + vs. -
Statins, n (%)	72.2	36.9	59.0	62.3	56.5	< 0.0001
Platelet aggregation inhibitors, n (%)	56.0	9.8	15.1	15.3	14.9	0.3312
Antihypertensive drugs						
ACEi, n (%)	39.0	26.8	61.5	62.3	61.0	< 0.0001
ARBs, n (%)	60.9	16.7	38.5	37.8	39.0	0.0385
MRAs, n (%)	**n.a. / 0**	4.6	7.7	8.1	7.5	0.0742
Beta-blockers, n (%)	49.9	36.3	61.0	60.6	61.2	0.3640
Diuretics						
Loop diuretics	21.5	14.1	23.5	22.0	24.7	< 0.0001
Thiazide diuretics	24.2	4.6	9.2	10.5	8.2	< 0.0001
Calcium antagonists, n (%)	56.5	20.3	37.6	40.3	35.5	< 0.0001
Glucose lowering therapies, n (%)						
Insulin, n (%)	58.6	58.9	63.6	63.0	64.1	0.0744
Metformin, n (%)	58.0	38.0	43.7	50.4	38.6	< 0.0001
Acarbose, n (%)	5.0	0.4	0.5	0.6	0.5	0.3312
Sulfonylurea, n (%)	26.0	5.2	5.5	5.9	5.2	0.0150
DPP-4 inhibitors, n (%)	25.2	25.8	28.4	28.2	28.7	0.4469
GLP-1 agonists, n (%)	7.2	6.4	8.4	9.7	7.3	< 0.0001
SGLT2 inhibitors, n (%)	6.7	8.0	10.0	12.4	8.2	< 0.0001

FIDELITY [21] was a pooled analysis of FIDELIO-DKD and FIGARO-DKD [19, 20]

*ACEi* angiotensin converting enzyme inhibitor; *ARB* angiotensin receptor blocker; *DPP-4* dipeptidyl peptidase-4; *GLP-1* glucagon-like peptide-1; *MRA* mineralocorticoid receptor antagonist (eplerenone, spironolactone, finerenone); *RCT* randomized controlled trial; *SGLT2* sodium-glucose transport protein-2

^a^The difference between the DPV/DIVE total and the combined RCT ± groups is the results of 36,809 patients that did not receive either an ACEi or ARB at baseline

### Impact of the FIDELITY specific CKD criteria on patient selection

**Fig. 2:**
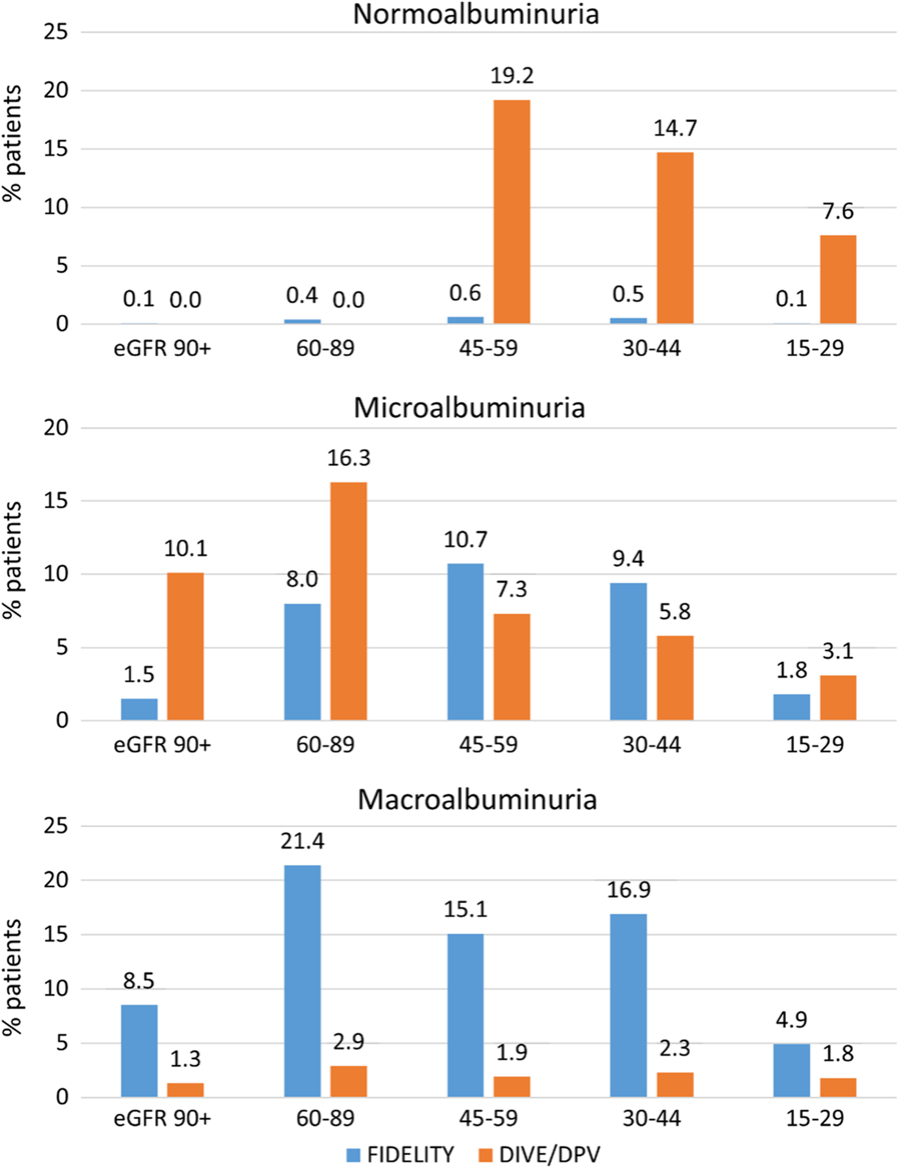
Patients’ CKD characteristics: comparison of DPV/DIVE population (n = 65,168) with FIDELITY clinical trial populations. The figure describes the proportion of patients in the FIDELITY and the DIVE/DPV analysis respectively by subgroups defined by albuminuria and eGFR. eFGR, estimated glomerular filtration rate

Based on the focus of albuminuric CKD patients in FIDELITY (Table 6), many of the 65,168 patients in DPV/DIVE were not represented by the RCTs because they were normoalbuminuric (urinary albumin-to-creatinine ratio [UACR] < 30 mg/g; 43.2%). In addition, patients with severely impaired kidney function (eGFR < 25 ml/min/1.73m^2^; 6.9%) and patients with only slight renal impairment (UACR 30–300 mg/g, normal eGFR [≥ 90 ml/min/1.73m^2^]; 10.1%) were not represented by the RCTs. This resulted in 59.2% of registry patients not being eligible for the RCTs based on the trials’ CKD definition alone, leaving 39.8% of the registry patients that had CKD compatible with the clinical trials’ CKD definition. As a consequence, a CKD severity shift was observed between patients in clinical practice versus patients considered for the RCTs (Fig. 2).

**Table 6:**
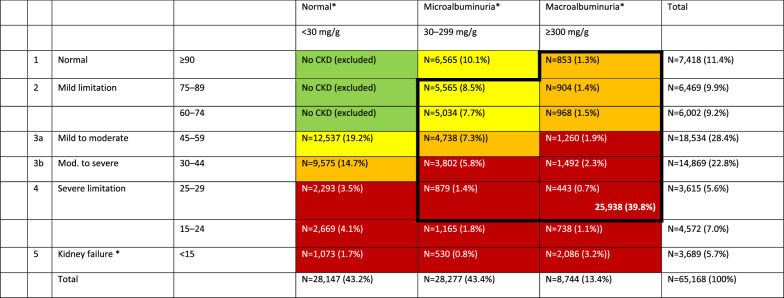
CKD characteristics based on eGFR and albuminuria in the DPV/ DIVE CKD population (based on [22, 28], n = 65,168)

Green, low risk (if no other markers of kidney disease, no CKD); Yellow, moderately increased risk; Orange, high risk; Red, very high risk. **Black border:** indicates patients eligible for inclusion in FIGARO-DKD or FIDELIO-DKD (n = 25.938; 39.8%) (see Table 1) based on CKD characteristics alone [17, 18]

*CKD* chronic kidney disease; *CKD-EPI* Chronic Kidney Disease Epidemiology Collaboration; *DKD* diabetic kidney disease; *eGFR* estimated glomerular filtration rate; *UACR* urinary albumin-to-creatinine ratio

^a^Or patients undergoing dialysis

### Impact of the trial‘s additional inclusion and exclusion criteria on patient selection

Considering the RCT’s requirement for baseline treatment with either ACEi or ARBs, the number of patients for analysis was reduced from 65,168 to 28,359. Overall, 56.5% of the registry patients were not treated with a RAS inhibitor although this is recommended by guidelines and, therefore, needs to be fulfilled by regulatory requirements during clinical trials. As this excluded patients based on clinical practice-specific decisions (e.g., prescription of RAS blockers) rather than on trial determined criteria, we further analyzed 28,359 of the 65,168 patients (43.5%) with ACEi/ARB pre-treatment to assess how all inclusion and exclusion criteria combined affected the representativeness of the trials for patients in clinical practice (Table 2). Overall, 12,322 patients fulfilled all the trials’ inclusion and exclusion criteria (43.5%), while 16,037 patients did not (56.5%).

The RCT-eligible patients were more often male (57.2 vs. 49.1%; p < 0.0001), had higher eGFR values (mean 60.1 vs. 48.6 mL/min/1.73 m^2^; p < 0.0001), and had higher rates of albuminuria (microalbuminuria [MAU] 75.1 vs. 19.9%; p < 0.0001) than patients not eligible for inclusion in the RCTs (Table 3, *columns 3 to 6*). Other differences were also mostly statistically significant, but likely of limited clinical relevancy. Furthermore, the comorbidity profile was quite consistent between RCT-eligible and non-eligible patients from clinical practice (Table 4, *columns 3 to 6*). Although some of the differences reached statistical significance, the absolute difference and, therefore, the potential clinical relevance, were small. Finally, the rates of statins (62.3 vs. 56.5%; p < 0.0001), calcium antagonists (40.3 vs. 35.5%; p < 0.0001), and the use of metformin (50.4 vs. 38.6%; p < 0.0001)/SGLT2-inihibitors (12.4 vs. 8.2%; p < 0.0001) was higher in RCT-eligible patients than in patients not eligible for the RCTs (*columns 4 and 5*).

To explore further potential differences in the risk profile between those eligible and non-eligible for the clinical trials, we computed the risk for cardiovascular mortality and progressive CKD as to Levey et al. [28] (Additional file 1: Table S1). We found that, while registry patients with low cardiovascular risk are not retained in the RCT ( +) or (−) population, patients in the RCT ( +) group had an increased cardiovascular risk as compared to the patients not eligible. The overall cardiovascular risk as determined by the SCORE-2 [31] was 9.8% in the RCT ( +) and 8.7% in the RCT (−) group (p < 0.0001) (Additional file 1: Table S2). This risk difference was mostly based on higher systolic blood pressure values, higher non-HDL cholesterol and slightly more smokers. A similar pattern was observed for the risk of progressive CKD (Additional file 1: Table S1). While RCT ( +) patients had a higher eGFR, more patients in the RCT (−) group had microalbuminuria more often (Table 3). Factors that perpetuate renal disease (Additional file 1: Table S3), were more common in the RCT ( +) group as exemplified by the rate of proteinuria (24.9 vs. 6.72%; p < 0.0001).

## Discussion

The principal findings of this analysis are as follows:

- 1) As expected, there was a noteworthy difference between patients included into the combined RCTs and those seen and treated in clinical practice; in particular with respect to a higher age, more female patients being treated, the comorbid CV disease profile, the lower rate of albuminuria, and the rate of appropriate ACEi/ARB drug use, which was rather low in clinical practice.
- 2) More specifically, based on the focus on CKD grade 1/2 to 4 with albuminuria in the RCTs, more than half of the patients with CKD in clinical practice (59.2%), namely normoalbuminuric patients, patients with severely impaired kidney function (eGFR < 25 ml/min/1.73m^2^), and patients with only slight renal impairment (moderate albuminuria and normal eGFR) were not covered by the finerenone clinical trial population.
- 3) Many of the patients in clinical practice with CKD (56.5%) were not pre-treated with ACEi or ARBs, although guidelines clearly recommend such treatment. For the RCTs, a run-in phase was conducted to adjust all patients to the individually highest tolerated in-label dose of ACEi or ARB. At baseline, all RCT-patients were treated with either an ACEi or an ARB. Therefore, the registry patients without RAS-inhibition were not covered by the RCTs. The application of the full set of inclusion and exclusion criteria to patients actually receiving ACEi/ARBs at baseline left 43.5% of patients that would have been included in the finerenone RCTs. Patients that would have been excluded from the RCTs were more often female, had lower eGFR values, lower rates of albuminuria and received less metformin/less SGLT2-inhibitors than RCT-eligible patients.
- 4) As a side-result, there was a clear undertreatment of CKD patients in clinical practice compared to the guideline recommendations.

### Comparison of CKD patients in the trials vs. clinical practice

As patients in RCTs need, by authority request, to fulfil all guideline requirements to investigate the effect of the new drug on top of optimized standard of care treatment, a clear difference has been expected for the comparison between RCT-patients and clinical practice. It is well known that patients in clinical trials tend to be younger and represent a higher proportion of male patients, as was observed in the present analysis [33–35]. In recent RCTs, more high-risk patients are included to show a relevant clinical benefit and to shorten study duration. Nonetheless, some differences were noteworthy and require further discussion: (1) Diabetic retinopathy as a comorbid condition was diagnosed more often in the clinical trial population. Diabetic retinopathy was low and comparable in DPV/DIVE patients with or without RAS blocking agents and after applying the RCTs’ inclusion and exclusion criteria to these patients. Because retinopathy was part of the inclusion criteria of FIDELIO-DKD, it can be assumed that its investigation was mandated by the study protocol at or prior to baseline, resulting in more patients being diagnosed and less patients overlooked. Furthermore, it appears that retinopathy is less well investigated and documented in clinical practice, especially in its early stages [36]. (2) On the contrary, diabetic neuropathy was more often diagnosed in the registry population than in the RCT patients. Although there was no specific exclusion criterion for patients with neuropathy, these patients may only rarely have been considered candidates for inclusion in the finerenone RCTs by the trial physicians based on reasons not documented. (3) For the RCTs, patients with a clinical diagnosis of chronic heart failure with reduced ejection fraction and persistent symptoms (New York Heart Association class II–IV) were excluded, as there is a clear guideline recommendation for an MRA-treatment; hence inclusion in a placebo-controlled MRA study is ethically unacceptable. The 7.5% of the patients with heart failure in the RCT are likely to represent patients with preserved ejection fraction, which makes up about half of all patients with heart failure [37]. In the registry we were not able to discriminate heart failure patients with preserved or reduced ejection fraction and, therefore, it appears reasonable to expect a higher rate of heart failure (12.3%) in the registry population than in the RCTs. (4) The use of CV and anti-diabetic drug therapy was increased in the RCT-patients compared to clinical practice, where a clear undertreatment was seen. The increased rate of treatment in the RCT population on the one hand results from the specification of the optimized guideline-conformant background therapy and, on the other hand, it reflects the increased attention and optimization efforts seen in RCTs [38]. A potential further contributor to the apparent discrepancy is a potential underreporting of CV drug therapy in the treatment of diabetes specialists, as the drugs may be prescribed by the treating cardiologist and not fully recorded in the diabetologist’s file. (5) Finally, patients in the registry had a lower eGFR and were more likely to be normoalbuminuric, which is a result of the focus on CKD patients with albuminuria in the RCTs as already outlined and will be discussed below.

### Exclusion of CKD patients based on the focus on CKD patients with albuminuria

The CKD definition commonly used today includes patients with a reduced eGFR (eGFR < 60 ml/min/1.73m^2^) with or without albuminuria. In comparison, the FILEDITY-population includes albuminuric patients only, as did most other RCTs in CKD-patients that have been published (DAPA-CKD, CREDENCE). The RCTs focus on albuminuric high-risk CKD patients for kidney and CV events [12, 39, 40] and a recent comparison of the trial population with finerenone eligible patients from the US [41] based on the NHANES data exactly described this picture: the US population had fewer individuals with severe albuminuria (> 300 mg/g) and more individuals with moderately elevated albuminuria (30–300 mg/g). In addition, it follows a classic diagnosis of diabetic nephropathy, which is based on the presence of persistent proteinuria, slowly evolving from a stage of MAU. Macroalbuminuria is then followed by a progressive decline in kidney function [42, 43]. While this so-called Mogensen sequence is still an important reference in type 1 diabetes, non-albuminuric renal impairment and progressive renal decline have more recently received increasing attention [44]. Pugliese et al. estimated that, among patients with T2DM, 50–65% have no CKD, 20–30% have albuminuria alone (but without a decline in the eGFR), and 15–25% have reduced eGFR. Of the latter subgroup with a reduced eGFR, about half of the patients present without relevant albuminuria, meaning that the other half of patients have reduced eGFR, but albuminuria [44]. This is in line with our own data; adding up eGFR stages 3 to 5 (n = 45,279) and putting these numbers into relation to those with eGFR stages 3 to 5 but without albuminuria (n = 28,147), we report a rate of 62.2%.

Similar to the present finding, patients with normoalbuminuric kidney disease were also excluded in the SGLT-2 inhibitor clinical trials CREDENCE [13, 45] and DAPA-CKD [46]. CREDENCE (Canagliflozin 100 mg/day) included patients with an eGFR 30 to 90 ml/min/1.73m^2^ and urinary albumin excretion of between 300–5000 mg/g [13, 45]. DAPA-CKD documented the effect of 5 or 10 mg/day of dapagliflozin in patients with an eGFR 25–75 ml/min/1.73m^2^ and urinary albumin excretion of between 200–5000 mg/g [46]. The latest SGLT-2 inhibitor trial, EMPA-KIDNEY (Empagliflozin 10 mg/day), considered patients with eGFR 20–45 *OR* eGFR 45–90 ml/min/1.73m^2^ with at least 200 mg/g of albuminuria [47]. Further to the above-mentioned CREDENCE and DAPA-CKD trials, normoalbuminuric patients with a severe decline of the eGFR (20–45 ml/min/1.73m^2^) were also documented in EMPA-KIDNEY. While the trial also included patients without diabetes and, therefore, cannot be directly compared to the results of our registry, it is the first to cover patients with normoalbuminuric CKD.

Given that normoalbuminuric CKD appears to be frequent with 43.2% of the patients in our clinical practice cohort with an eGFR below 60 ml/min/1.73m^2^, clinical trials studying the effects of drug treatment in this patient population, either with finerenone or SGLT2 inhibitors, are strongly desired.

### Exclusion of patients based on the lack of ACEi/ARBs use

When selecting patients for the present analysis, we arrived at a number of 65,168 patients with CKD in the DPV/DIVE cohort. Of these, only 28,359 patients were prescribed either ACEi or ARBs, leaving 56.5% without any blockade of RAS despite CKD although guidelines recommend their use [12]. This probably is due to the inclusion of patients with either early or normoalbuminuric CKD. The rate is higher than reported rates of 36% non-prescription in moderate to severe CKD [48]. It further agrees with a previously published study from the US which looked into the use of antihypertensive drugs in patients with CKD [49]. Less than one half of the participants with CKD in the NHANES were using antihypertensive drugs. While beta-blockers were the most commonly used, ARBs were the least used antihypertensive agents among participants with CKD. Age (≥ 70 years), awareness of hypertension or diabetes, and higher stage of CKD were associated with an increased likelihood of antihypertensive drug use among participants with CKD. In our cohort, patients had a mean age of 73 years, 22.0% were not hypertensive based on a threshold of 140/90 mmHg, 31.3% had an eGFR of ≥ 60 mL/min/1.73 m^2^, and 43.2% of the patient had albuminuria not reaching 30 mg/g. Taken together the potential lack of coverage of the finerenone trials for clinical practice patients was mostly based on the lack of ACEi/ARB prescription in the registry patient population.

### Exclusion of patients based on further trial specific criteria

The application of the full set of inclusion and exclusion criteria to patients actually receiving ACEi/ARBs at baseline left 43.5% of patients who would have been included in the finerenone RCTs. While the selection criteria ensure guideline-consistency, comparability of patients and patients’ safety in the clinical trials, they also exclude many patients that need further treatment in clinical practice. Patients that would have been excluded from the RCTs were slightly older, more often female, had lower eGFR values, substantially lower rates of albuminuria and received less SGLT2-inhibitors than eligible patients. As older people with CKD are also less likely to have albuminuria than younger people [50], it is possible that the lower frequency of albuminuria in the RCT-ineligible population compared with the RCT-eligible population could in part relate to these criteria.

Moreover, we observed and increased cardiovascular and renal risk in the registry population eligible for the clinical trials than in patients not eligible. This was based on a variety of specific risk factors such as systolic blood pressure values, non-HDL cholesterol levels and smoking. Also, the level of proteinuria was, as already outlined above, increased in RCT eligible patients.

The pattern of CV and diabetes-related drug therapy differed substantially between the finerenone RCTs and clinical practice, mainly based on the undertreatment with RAS blockers of the registry patients. However, if patients not treated with RAS blockers are excluded from the comparison, treatment patterns did not differ much. Furthermore, there was an apparent undertreatment with SGLT-2i and GLP-1RA despite the available evidence to support their use. It was higher though in the RCT eligible registry patients than in the clinical trials: GLP-1RA 9.7% vs. 7.2% and SGLT-2i 12.4% vs. 6.7%. Several potential factors may contribute to the low prescription rate, including prescriber hesitancy, treatment inertia and increased drug costs [51]. A lower use of statins, platelet aggregation inhibitors, ARBs, and calcium channel blockers were the most prominent differences. While the lack of platelet aggregation inhibitor use may be related to a lower prevalence of CAD in the clinical practice setting, all other treatments were intensified in the finerenone RCTs, due to guideline and authority recommendation and by a closer surveillance and optimized treatment when entering the trial.

### Strengths and limitations

Major strengths of the current analysis are the large number of patients and the good quality of the data recorded in the DPV/DIVE databases. Nonetheless, not all information pertinent to the RCT selection criteria were available for all patients. For example, during the initial search in the entire DPV/DIVE database (n = 718,138), 102,439 patients did not have an eGFR value available (vs. 51,540 who were recorded as having no CKD), and it is possible that some of these patients might have been suitable for inclusion in the analysis if these data had been available. Furthermore, we were not able to discriminate heart failure with or without preserved ejection fraction and, as such, we were not able to fully resemble the trial inclusion and exclusion criteria in this regard. Finally, the clinical effectiveness of finerenone and its safety in clinical practice could not be evaluated as it was not registered at the time of the analysis. It should be kept in mind that p-values are based on large patient numbers and not all significant differences may have clinical relevancy.

## Conclusions

The main findings of the present analysis are that certain patient subgroups, especially non-albuminuric CKD-patients, a common group in clinical practice, were not included in the finerenone RCTs. Furthermore, the registry data showed an undertreatment of CKD patients with RAS blockers in clinical practice, although guidelines clearly recommend such treatment. These limitations result in the study population being only partially comparable to clinical practice as data on finerenone are, therefore, not available for certain common patient groups. Further research into patients with normoalbuminuric CKD and a wider prescription of RAS blocking agents for patients with CKD in clinical practice appears warranted.

## Supplementary Information

**Additional file 1: Table S1**. Cardiovascular and renal risk [28]. **Table S2**. Determinants of cardiovascular risk [31]. **Table S3**. Renal disease—perpetuating factors* [32].

## Data Availability

The datasets generated and analyzed during the current study are not publicly available due to data privacy but aggregated data may be available from the corresponding author on reasonable request.
