# Supplementary material for: An analysis of DPV and DIVE registry patients with chronic kidney disease according to the finerenone phase III clinical trial selection criteria
Source: Cardiovasc Diabetol. 2023 May 8;22:108. doi: 10.1186/s12933-023-01840-5 (PMC10169333; doi:10.1186/s12933-023-01840-5)
Supplement: Supplementary file 1 — Additional file 1: Table S1. Cardiovascular and renal risk [28]. Table S2. Determinants of cardiovascular risk [31]. Table S3. Renal disease—perpetuating factors* [32]. [file 12933_2023_1840_MOESM1_ESM.docx]

**Table S1:** Cardiovascular and renal risk [28]

|  | RCT (+) | RCT (-) | p-value* |
| --- | --- | --- | --- |
|  | n=12,322 | n=16,037 |  |
| **Cardiovascular mortality, %** |  |  |  |
| Low risk | 0.00 | 0.01 | 0.3620 |
| Moderately increased risk | 39.3 | 49.0 | <0.0001 |
| High risk | 39.1 | 31.0 | <0.0001 |
| Very high risk | 21.6 | 20.0 | 0.0017 |
| **Progressive CKD, %** |  |  |  |
| Low risk | 0.00 | 16.6 | <0.0001 |
| Moderately increased risk | 41.8 | 0.01 | <0.0001 |
| High risk | 10.0 | 39.0 | <0.0001 |
| Very high risk | 48.3 | 44.4 | <0.0001 |

*Legend:* * p-values are adjusted with the False Discovery Rate

**Table S2:** Determinants of cardiovascular risk [31]

|  | RCT (+) | RCT (-) | p-value* |
| --- | --- | --- | --- |
|  | n=12,322 | n=16,037 |  |
| SCORE 2 CV risk, % | 9.8 ± 4.1 | 8.7 ± 4.0 | <0.0001 |
| Age, years |  |  |  |
| 40-44 | 0.72 | 0.99 | 0.0211 |
| 45-49 | 1.50 | 2.22 | <0.0001 |
| 50-54 | 3.82 | 3.66 | 0.5068 |
| 55-59 | 6.40 | 6.03 | 0.2239 |
| 60-64 | 9.07 | 8.19 | 0.0129 |
| 65-69 | 13.53 | 10.53 | <0.0001 |
| Systolic blood pressure, mmHg |  |  |  |
| 100-119, % | 9.76 | 13.09 | <0.0001 |
| 120-139, % | 41.62 | 44.14 | <0.0001 |
| 140-159, % | 32.19 | 28.27 | <0.0001 |
| 160-179, % | 10.67 | 8.24 | <0.0001 |
| Non-HDL cholesterol, mmol/L |  |  |  |
| 3.0-3.9 | 4.58 | 4.68 | 0.6747 |
| 4.0-4.9 | 9.25 | 8.57 | 0.0573 |
| 5.0-5.9 | 12.56 | 10.81 | <0.0001 |
| 6.0-6.9 | 13.73 | 11.35 | <0.0001 |
| Smoking status, yes, % | 9.64 | 8.64 | 0.0056 |

*Legend:* * p-values are adjusted with the False Discovery Rate

**Table S3:** Renal disease - perpetuating factors* [32]

|  | RCT (+) | RCT (-) | p-value** |
| --- | --- | --- | --- |
|  | n=12,322 | n=16,037 |  |
| Proteinuria (Macroalbuminuria) | 24.9 | 6.72 | <0.0001 |
| SBP >130 mmHg | 138.7±19.2 | 135.0±18.8 | <0.0001 |
| Obesity (BMI ≥30 kg/m^2^) | 53.8 | 53.1 | 0.3051 |
| Anemia | 5.0 | 7.7 | <0.0001 |
| Dyslipidemia | 92.0 | 91.2 | 0.0282 |
| Smoking | 9.6 | 8.6 | 0.0056 |
| Cardiovascular disease |  |  |  |
| CAD | 13.2 | 14.5 | 0.0029 |
| PAD | 27.5 | 27.0 | 0.3995 |
| Myocardial infarction | 12.3 | 13.5 | 0.0049 |
| Stroke | 11.6 | 11.7 | 0.8806 |
| CABG | 1.8 | 2.2 | 0.0069 |
| Heart failure | 15.3 | 17.7 | <0.0001 |

*Legend:* * data on African-American race, number of nephrons, dietary protein intake and nephrotoxins not available from the DIVE / DPV datasets; **p-values are adjusted with the False Discovery Rate
